# Regional gradients in intraspecific seed mass variation are associated with species biotic attributes and niche breadth

**DOI:** 10.1093/aobpla/plac013

**Published:** 2022-03-30

**Authors:** Xiaomei Kang, Jieyang Zhou, Abuman, Yanjun Liu, Shiting Zhang, Wei Liu, Haiyan Bu, Wei Qi

**Affiliations:** 1 State Key Laboratory of Grassland Agro-ecosystems, College of Ecology, Lanzhou University, Lanzhou 730000, China; 2 Gansu Provincial Extension Station of Grassland Techniques, Lanzhou 730000, China

**Keywords:** Intraspecific trait variation, life form, light niche, niche breadth, Qinghai–Tibet Plateau, seed dispersal mode, seed mass, thermal niche

## Abstract

Quantifying intraspecific trait variation (ITV) is crucial for understanding species local adaptation and regional distribution. Intraspecific seed mass variation (ITVsm) is expected to vary with environmental gradients or co-vary with related biotic attributes, but these relationships are not well known in a multispecies space. We performed interspecific and phylogenetic analyses to evaluate the relative power of three species biotic attributes and four niche breadth traits in explaining ITVsm variation for 434 eastern Qinghai–Tibetan species. We showed a positive relationship between species’ ITVsm and their niche breadth in the light, moisture and disturbance dimensions, supporting the idea that high ITV allows species to match their traits to different habitat conditions and thus to distribute in a wide range of environments. However, we did find significant direct effect of species’ thermal niche on individual seed mass variation. Meanwhile, we showed significant effects of seed dispersal mode, but not of life form and pollination type, on ITVsm. This suggests that the covariation or co-evolution between seed and disperser was related to the pattern and magnitude of ITVsm, but not to plant lifespan, the quality and allocation pattern of available resources and the availability of pollination vector. Lastly, all multivariate models showed a significant combined contribution of species’ biotic attributes and niche breadth to their ITVsm, implying that intrinsic biotic limitations and extrinsic abiotic pressures may operate simultaneously in controlling regional-scale intraspecific seed development.

## Teaser

We evaluated the extent to which species biotic attributes and niche breadth affected their intraspecific variation in seed mass (ITVsm). Results showed a significant directly positive effect of species niche breadth in the light, moisture and disturbance dimensions, but not in the thermal dimension, on their ITVsm. Seed dispersal mode, rather than life form and pollination type, was the only significant biotic predictors of ITVsm. Lastly, our multivariate models suggest that intrinsic biotic limitations, especially the covariation or co-evolution between seed and disperser, and extrinsic abiotic conditions, especially seedlings’ adaptation to habitat pressures, operate simultaneously in controlling regional-scale ITVsm.

## Introduction

Reproductive success of most plants depends on their seeds (Baskin and Baskin 2014; Igea *et al.* 2017). Seed mass generally indicates the substantial nutrient reserves in seed, and thus is considered to be pivotal to early plant survival (Moles 2018; Vandelook *et al.* 2018; Kang *et al.* 2021). Because of the limitation of available resources for seed development in a growing season, plants can produce either many small seeds to increase an opportunity of distributing widespread or being dispersed to more habitats or sites (Leishman and Westoby 1994; Igea *et al.* 2017), or few large seeds with more resources to increase a chance of good early seedling establishment (Moles *et al.* 2005; Moles and Westoby 2006; Baskin and Baskin 2014; Moles 2018). Consequently, different populations of a species under contrasting environmental conditions may exhibit different seed mass due to diverging selective pressures (Veloso *et al.* 2017; Fricke *et al.* 2019). The previous studies have reported that the magnitude of intraspecific trait variation (ITV) in response to environmental gradients can vary strongly among species with different biotic attributes or evolutionary history (Herz *et al.* 2017; Zhang *et al.* 2020; Westerband *et al.* 2021), suggesting that species intrinsic biotic attributes may contribute at least partly to the pattern of ITV. Thus, intraspecific seed mass variation (ITVsm) is expected to be affected by both the environmental and biotic factors (Sack 2004; Fricke *et al.* 2019).

Due to a tight trait–environment relationship, it seems intuitive to think that the species’ niche breadth is positively correlated with their ITV (Violle and Jiang 2009; Violle *et al.* 2012; Sides *et al.* 2014; Fajardo and Siefert 2018, 2019); that is, a large intraspecific variation translates into a strong capacity of species to prevail in a wide range of environments. Hence, plant species can occur over relatively broad spatial scales because high ITV allows them to match their traits to different environmental conditions (Violle *et al.* 2012; Sides *et al.* 2014; Fajardo and Siefert 2019). The extent of ITVsm is likely to be positively related to the species’ niche breadth. Among niche dimensions, the species’ light and moisture niches, representing their adaption to the light and moisture conditions in a habitat, can be considered first in examining ITVsm because these habitat conditions directly affect the early-stage plant life-history traits related to seed mass, e.g. seed germination and seedling performance, both in within-species studies (Baskin and Baskin 2014; Veloso *et al.* 2017) and between-species researches (Leishman and Westoby 1994; Vandelook *et al.* 2018). Normally, especially in most large-scale interspecific studies, large seeds are common in low-light and low-moisture conditions to strengthen seedling growth and survival under shade and drought habitats (Sack 2004; Moles and Westoby 2006; Igea *et al.* 2017; Vandelook *et al.* 2018). However, the low-light or low-moisture conditions in some regions, representing suboptimal resource availability for reproductive output and seed development, may select for small-seed species or populations (Sánchez-Gómez *et al.* 2006; Moles 2018; Kang *et al.* 2021). These opposite forces may result in an unclear species niche–seed mass relationship. Species thermal niche, generally defined by local thermal conditions or temperature levels, has a dominant effect on species distributions and individual plant growth (Fernández-Pascual *et al.* 2019; Löffler and Pape 2020). Hence, it is easy to deduce that species with the wide thermal niche breadth can have large among-population variation in individual size as the response to the variation in local thermal energy, indirectly causing large ITVsm. Moreover, disturbance is often regarded as an environmental factor that is proven to restrict the survival of large-seed individuals of a species in high-disturbance, and of small-seed ones in low-disturbance surroundings (Wu *et al.* 2015; Phartyal *et al.* 2020). Thus, the species disturbance niche breadth is expected to correlate positively with ITVsm.

Among the intrinsic factors possibly associated with ITVsm, seed dispersal mode and life form can be considered first because their evolutionary divergences have been thought to be the drivers of the geographic distribution and evolution in seed mass. For example, some interspecific studies have found that the divergence of life form, especially it of woody and herbaceous plants, is the primary factor affecting global seed mass divergence (Moles *et al.* 2005; Moles and Westoby 2006), whereas others have confirmed the evolution/divergence of seed dispersal mode, especially in case of vertebrate dispersal, as the main force of global distribution and evolution in seed mass (Tiffney 2004; Zhang *et al.* 2020). Compared with most species whose seed mass is controlled mainly by genetic variation and available resources (Venable 1992; Paul-Victor and Turnbull 2009; Li *et al.* 2019), the mass of anemochorous and zoochorous seeds is also affected by seed dispersers (e.g. wind and animals; Butler *et al.* 2007; Zhang *et al.* 2020). Thus, we can expect that these seeds show a smaller intraspecific variation than autochorous seeds because the mass variation of the former needs to match structurally the size of dispersal appendages such as wings, hairs, pappus, hooks, spines, juicy aril or flesh (Lord *et al.* 1997; Eriksson 2016; Kang *et al.* 2021). We also expect woody species to have smaller ITVsm than herbaceous species because the former have larger individual biomass and the ability to flower early (Bolmgren and Cowan 2008; Du *et al.* 2020), which may reduce the dependence of their seed development on the growing season, available resources and (or) habitat conditions (Butler *et al.* 2007; Qi *et al.* 2015). In addition, because of the significant difference among pollination types in their pollination efficiency in different environmental conditions, and the significant effect of pollination efficiency on species reproductive success and individual seed number (Totland and Eide 1999; Knight *et al.* 2005), we can expect significant influences of pollination system on ITVsm through a seed size/number trade-off (Zhang *et al.* 2020).

Relative to most of the other regions in the world, the Qinghai–Tibet Plateau (QTP) varies significantly in climate parameters (Kang *et al.* 2010). The dramatic climate changes accelerate the alterations in regional or local vegetation features and environmental conditions, which, ultimately, force a rapid variation in plant traits in response to these changes. Thus, QTP is the ideal place to evaluate the current or forecast the future effects of environmental variations on the species distribution and variation in plant traits (Favre *et al.* 2015). Here, using a database of 434 generalist angiosperm species in the eastern part of QTP, we focused on the main factors, including three species attributes and four niche breadth traits, potentially contributing to ITVsm in the regional flora. This is, to our knowledge, the first study to explicitly disentangle the various abiotic and biotic drivers of the intraspecific variation in a trait across species of a regional flora by using a large database with a wide environmental span. The study will help reconcile two sides of argument caused mainly by previous few-species or few-factor researches whether co-varying with biotic attributes or plastic response to environmental gradients is the determinant of regional patterns of intraspecific trait variation. Specifically, we addressed the following questions:

1) Do species with large niche breadth have large ITVsm? What are the major niche factors that correlate with ITVsm: temperature, light, moisture and/or disturbance?
2) Does the extent of ITVsm vary significantly among species with different life forms, seed dispersal modes or pollination types?

To answer these questions, we determined the relative importance of intrinsic biotic attributes and niche breadth traits in ITVsm by using both interspecific and phylogenetic analyses. Based on these analyses, we expected (i) a positive ITVsm–niche breadth correlation both with and without controlling for phylogeny and (ii) a smaller ITVsm for woody and zoochorous species than for others.

## Materials and Methods

### Study site

The study area is located on the north-east verge of Tibet Plateau (100°50ʹ–103°40ʹE, 33°50ʹ–35°70ʹN; 1700–4100 m a.s.l.), and belong to a transitional area of Qinghai–Tibetan Plateau, Qinling mountain and Loess Plateau. This region spans a large climate range, with a mean annual temperature from −1.7 to 13.2 °C, with mean annual precipitation from 343 to 709 mm.

### Species studied

Through 16 years’ (2001–16) seed collecting and field investigation, we are able to gather a large and unprecedented comprehensive data set of a flora (~1580 species and 9800 populations; part of data are unpublished, while other data are showed in Qi *et al.* 2014, 2015 and Kang *et al.* 2021). For 417 populations (belonging to 352 species, being included in the analysis) for which seeds were sampled for no fewer than 3 years, we did not find significant seed mass difference among years (results not shown), suggesting that the temporal effect (i.e. the effect caused by annual changes in climatic conditions, such as temperature and precipitation) on ITVsm is weak. Therefore, we did not need to consider annual variation of seed mass in this study. Finally, we selected a large data subset of 434 species for which seed mass had been measured in no fewer than seven populations each (altogether 4769 populations) **[see** **Supporting Information—Supplementary Material** **and** **Appendix S3****]**. We used this species selection criterion because (i) the selected species had a large distribution range (i.e. regional widespread species) and (ii) the other species sample size was considered too low for testing intraspecific trait variation reliably. These species cover a wide taxonomic range, being derived from 199 genera and 58 families which based on Angiosperm Phylogeny Group IV system.

### Data source

Seed mass was defined as the weight of the embryo and endosperm, plus the seed coat. Other structures contributing to dispersal were not included as part of the seed. Seeds (*n* = 100 whenever possible) from each population were air-dried weighed three times, and the mean weights were used in further calculation (Qi *et al.* 2014, 2015; Kang *et al.* 2021). For every species, we evaluated three intrinsic biotic attributes, including life form (being classified as annual, herbaceous perennial and woody perennial), seed dispersal mode (autochory, anemochory and zoochory (ectozoochory and endozoochory)) and pollination type (anemophily and entomophily). For every population, we recorded its temperature regime, disturbance degree of the habitat as well as light and moisture levels to determine its niche positions in the light, moisture, disturbance and thermal dimensions (five levels for each niche dimension) **[see** **Supporting Information—Appendix S1****]**. Specifically, we classified population’s thermal niche position based on the thermal level (i.e. thermal climatic zone; including alpine, cold-subalpine, warm-subalpine, cold-temperature and warm-temperature zone) it survives. Light/moisture/disturbance niche of a population was defined according to the light/moisture/disturbance level of habitat where more than its 70 % individuals (or more than its 70 % individuals that were collected seeds) occupied. In order to meet the requirement of calculating species niche breadth (each population having only one niche level in each dimension), we excluded the population whose <70 % individuals lived in one niche level. The details of the category and the measurement of biotic attributes of every species and the niche positions of every population were described in **Supporting Information—Appendix S1**.

### Quantifying ITVsm and species niche breadth

For each species, we quantified its ITVsm by using the coefficient of variation (CV) among populations **[see** **Supporting Information—Supplementary Material****]**. The CV was chosen as an index of relative rather than absolute variation, thus allowing direct comparison among species with different mean seed mass. The CV values of among-population seed mass variation were denoted CVsm. For each species, we calculated niche breadth in each dimension (light, moisture, thermal and disturbance) by using Levins’ *B* index based on the niche positions of every population (Papacostas and Freestone 2016). Levins’ *B* of every species is calculated as: 1/∑*pi*^2^, where *pi* is the proportion of niche position (i.e. light, moisture, thermal or disturbance level) *i*. The values of this index range from 1 to *N* (number of niche positions), and large values indicate a wider niche breadth.

### Statistical analyses

All analyses below were carried out using R version 4.0.2 or SPSS 24.0 unless stated otherwise.

#### Interspecific analysis.

 Firstly, we evaluated the single effect of species biotic attributes (seed dispersal mode, life form and pollination types; as categorical variables) and niche breadth traits (light, moisture, thermal and disturbance niche breadth; as continuous variables) on CVsm by using one-way ANOVA and simple linear regression, respectively. Then, we performed generalized linear models (GLM) to test for the integrative effect of species biotic traits (as fixed factors) and niche breadth traits (as covariate) on CVsm. We also performed phylogenetic analysis (PA), but current implementations of the standard PA cannot deal with unordered categorical independent variables. Thus, for a direct comparison of PA and GLM, life form was treated as two binary variables in these analyses: xylophyta (woody/herbaceous: 1/0) and lifespan (perennial/annual: 1/0), and seed dispersal mode as: anemochory (yes/no: 1/0) and zoochory (yes/no: 1/0). In addition, entomophily/anemophily was coded as 1/0 in PA and GLM.

To better assess the factors affecting CVsm, we carried out regression tree models with species all biotic attributes and niche breadth traits as predictor variables. The trees can deal with non-linear and hierarchical relationships and provide reliable parameter estimates because the method guards against the elimination of variables that are good predictors of the response but are correlated with other predictors (Pyšek *et al.* 2012). The trees were constructed in CART by binary recursive partitioning, using the default ‘Gini’ impurity measure with a minimum child node of 10 species. Tenfold cross-validation was used to improve the accuracy of the tree.

#### Phylogenetic analys*is.*

Phylogenetic analysis is an effective method to assess whether interspecific relationship is independent of species’ phylogeny. The comparison between interspecies and phylogenetic analyses can also help assess whether present-day trait relationship is a consistent pattern during trait evolutionary divergence. The analyses need a working evolutionary tree, which was obtained from an online website (http://phylodiversity.net) based on a comprehensive angiosperm phylogeny from Zanne *et al.* (2014). Prior to analyses, we examined the phylogenetic signal strength by estimating Pagel’s *λ* for CVsm and all related biotic and niche traits in the R package ‘phytools’ (Revell 2012). We used a maximum likelihood framework to estimate the parameter *λ*, which can vary from 0 (no influence of phylogeny) to 1 (maximum phylogenetic influence). The package also provides *P*-values by performing a likelihood ratio test against the null hypothesis that *λ* = 0 **[see** **Supporting Information—Table S1****]**. In phylogenetic analyses, the effect of each of species biotic attributes (xylophyta, lifespan, anemochory, zoochory and pollination types; as binary variables) and niche breadth traits (light, moisture, thermal and disturbance niche breadth; as continuous variables) on CVsm was determined by using phylogenetically independent contrasts (PICs) and phylogenetic regression (PR; a regression analysis on the PICs of each trait against its standard deviation (SD)), respectively. Phylogenetically independent contrasts were calculated using the ‘Analysis of Traits’ model in Phylocom (Webb *et al.* 2008); whereas PR (i.e. regression analysis of standardized contrasts (forced through the origin)) was performed using SMA (standardized major axis) analysis in R.

## Results

Across all species, the mean (± SD) of CVsm and light, moisture, thermal and disturbance niche breadth was 0.207 (0.070), 1.812 (0.479), 1.834 (0.449), 2.628 (0.681) and 1.958 (0.436), respectively. Meanwhile, phylogenetic signal of pollination type (*λ* = 0.999), xylophyta (*λ* = 0.999), anemochory (*λ* = 0.881) and zoochory (*λ* = 0.817) was significant and strong **[see** **Supporting Information—Table S1****]**, of lifespan (*λ* = 0.494), light niche breadth (*λ* = 0.444) and moisture niche breadth (*λ* = 0.279) was significant and weak, of CVsm (*λ* = 0.140) was marginally significant (0.05 < *P* < 0.1), but of disturbance and thermal niche breadth was non-significant (both *λ* < 0.001 and *P* = 1) **[see** **Supporting Information—Table S1****]**.

**Figure 1.**
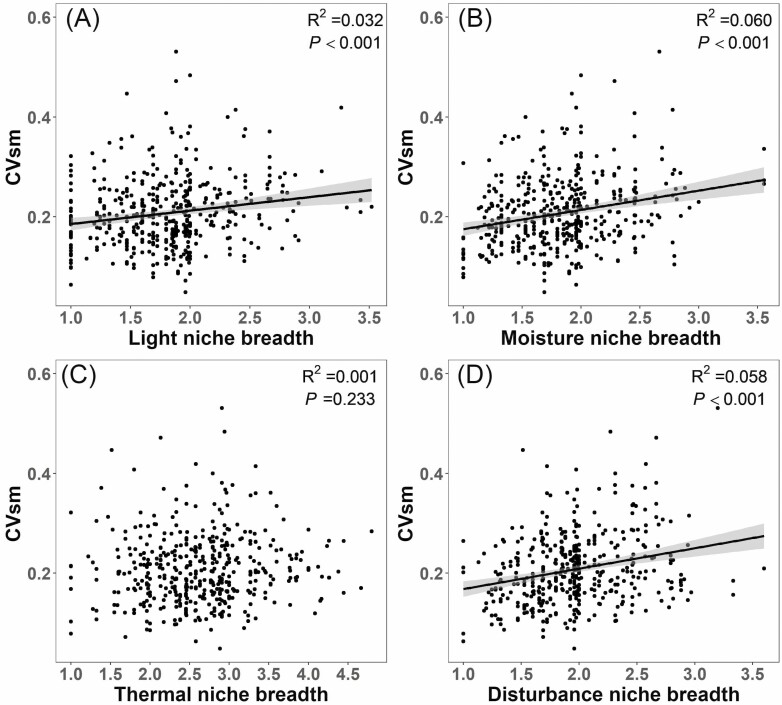
Linear relationship between CVsm (the coefficient of variation of among-population seed mass) and species niche breadth in light (A), moisture (B), thermal (C) and disturbance (D) dimensions. Regression lines are shown for significant relationships (*P* < 0.05).

**Figure 2.**
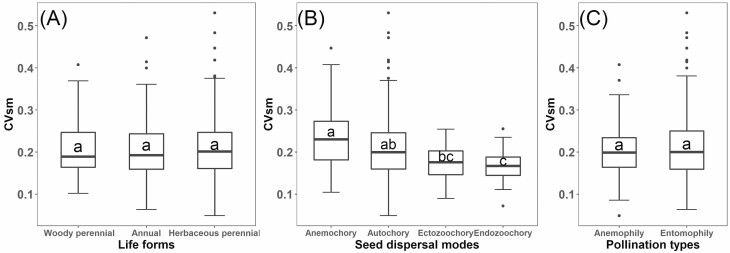
Box plot of the variation in CVsm (the coefficient of variation of among-population seed mass) with life forms (A), seed dispersal modes (B) or pollination types (C). The ends of the box represent the first and third quartiles and the middle line represents the median. The error bars indicates 1.5-fold the interquartile range. The different lowercase letters indicate significant differences of CVsm among life form, seed dispersal mode or pollination type.

**Figure 3.**
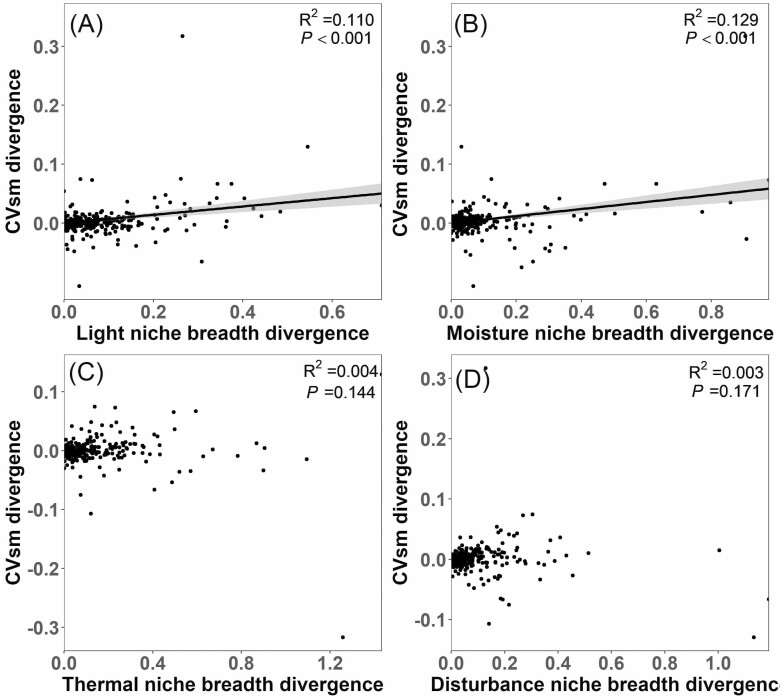
Linear relationship between divergence in CVsm (the coefficient of variation of among-population seed mass) and divergence in niche breadth in light (A), moisture (B), thermal (C) and disturbance (D) dimensions, respectively (*N* = 229 for all relationships). Regression lines are shown for significant relationships (*P* < 0.05).

The association of CVsm with niche breadth in light (Fig. 1A), moisture (Fig. 1B) and disturbance (Fig. 1D) dimensions was significantly positive, but was non-significant with thermal niche breadth (Fig. 1C). The CVsm was non-significantly different among species with different life forms (Fig. 2A) and pollination types (Fig. 2C), but varied significantly among species with different seed dispersal modes (Fig. 2B), with zoochorous (ectozoochorous and endozoochorous) species having smaller CVsm than others. The PR confirmed the significantly positive association of CVsm with light niche breadth (Fig. 3A) and moisture niche breadth (Fig. 3B) and the non-significant association of CVsm with thermal niche breadth (Fig. 3C), but did not confirm the positive CVsm–disturbance niche breadth relationship (Fig. 3D). Phylogenetically independent contrasts showed significantly lower divergence in CVsm for zoochory than other seed dispersal modes (paired *t*-test, the same below; *N* = 26, mean = −0.032, *P* = 0.010). In contrast, the divergence in CVsm was not affected significantly by xylophyta (*N* = 16, mean = −0.019, *P* = 0.343), lifespan (*N* = 36, mean = 0.001, *P* = 0.925), anemochory (*N* = 18, mean = 0.029, *P* = 0.063) or pollination types (*N* = 8, mean = 0.017, *P* = 0.476).

**Figure 4.**
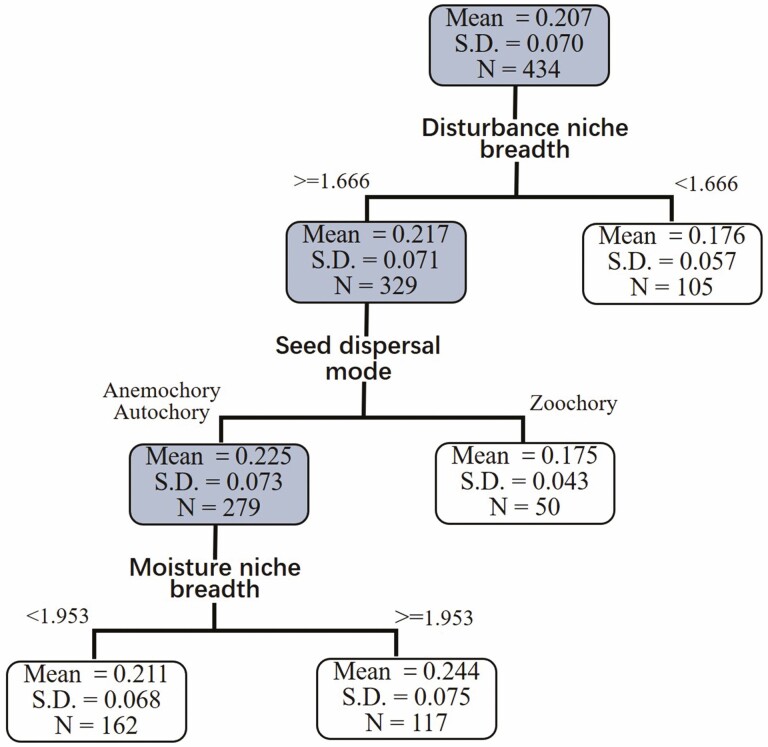
Results of the regression tree analysis of the relationship between CVsm (the coefficient of variation of among-population seed mass) and seven biotic or niche breadth predictors. In the decision of tree size, 10-fold cross-validations and a minimum child node of 10 sampling size are applied, and the Gini index is used as impurity.

Multivariate analysis (GLM) identified moisture niche breadth as the strongest factor influencing CVsm (Table 1), following by disturbance niche breadth, zoochory, anemochory and light niche breadth. In contrast, the effect of xylophyta, lifespan, pollination types and thermal niche breadth on CVsm was not significant (Table 1). In the regression tree model, disturbance niche breadth was the first dividing criterion for exploring CVsm, in which species with high disturbance niche breadth had high CVsm (Fig. 4). In the second level of the regression tree, seed dispersal model was the dividing criterion in the group of species with high disturbance niche breadth, in which the zoochorous species subgroup had low CVsm. For the subgroup of non-zoochorous species with high disturbance niche breadth, species with low moisture niche breadth had low CVsm (Fig. 4).

**Table 1.** Results from GLM of various predictors on the coefficient of variation of intraspecific seed mass (CVsm). For binary (xylophyta, lifespan, anemochory, zoochory and pollination type) and continuous (light niche breadth, moisture niche breadth, thermal niche breadth and disturbance niche breadth) predictors, column ‘B’ represented the mean CVsm difference between groups (group ‘1’ minus group ‘0’) and regression slope, respectively. %SS, percentage of total sum of squares explained. *P < 0.05, **P < 0.01 and ***P < 0.001, respectively.

Predictor	*B*	*F*	%SS
Models	Adjusted *R*^2^ = 0.184		
Xylophyta	0.017	1.91	0.4
Lifespan	−0.002	0.03	0.0
Anemochory	0.027	12.65***	2.9
Zoochory	−0.039	16.35***	3.6
Pollination type	0.000	0.00	0.0
Light niche breadth	0.022	10.80**	2.5
Moisture niche breadth	0.034	22.92***	5.0
Thermal niche breadth	−0.001	0.01	0.0
Disturbance niche breadth	0.032	18.14***	4.0

In addition, the validity of the above results was confirmed by similar trends in the variation of ITVsm when Gini coefficient value of among-population seed mass variation (GCsm) was used instead of CVsm in all of our analyses **[see** **Supporting Information—Appendix S2****]**.

## Discussion

The reasons for intraspecific variation in a certain plant trait are multiple; such variability arises from a combination of genetic variation, developmental instability and phenotypic plasticity due to environmental change, and thus, is expected to be influenced by abiotic and biotic factors (Violle *et al.* 2012; Sides *et al.* 2014; Vandelook *et al.* 2018; Fajardo and Siefert 2019; Fricke *et al.* 2019; Westerband *et al.* 2021). This is supported by our findings that the variation in ITVsm is associated with species biotic attributes and niche breadth. However, the effects of different biotic attributes or different dimensions of niche breadth of species on ITVsm were significantly different, suggesting that multiple mechanisms may operate simultaneously in governing seed development. Below, we discuss our findings as well as offer potential explanations for some of the unexpected results.

In congruent with the most findings and our first expectation, we showed that species with wide light and moisture niche breadths had high ITVsm, supporting the common hypothesis that high phenotypic plasticity or genotypic variability enhances species niche breadth by allowing species to express advantageous phenotypes or genotypes in a broad range of habitats (Violle *et al.* 2012; Sides *et al.* 2014; Fajardo and Siefert 2019). However, the interpretive force of these two niche traits to ITVsm was different, in which moisture niche breadth is the stronger predictor of ITVsm. The reason may be that habitat moisture conditions can affect intensely early-stage plant life-history processes such as seed germination and seedling survival or establishment. These processes are affected by seed mass to some extent (other seed traits, such as seed nutrient content and seed metabolic rate, can also be influential; Sack *et al.* 2004; Moles and Westoby 2006; Baskin and Baskin 2014; Vandelook *et al.* 2018). Thus, to get the local optima in these processes, species may develop different seed mass in response to the variation in the habitat moisture conditions. In contrast, the habitat light conditions (representing the amount of light resources available for plant growth) may affect mainly plant growth rate rather than seedling establishment, and thus, weakening the dependence of ITVsm on habitat light variation.

In contrast to our first expectation, the relationship between niche breadth thermal and ITVsm was not significant. In the present study, the thermal niche of species or population was defined according to the local thermal or temperature level, whereas the term is often used to represent the amount of thermal energy available for individual plant development. Thus, the non-significant relationship may imply that plant species respond to thermal gradients mainly by changing their individual size, total reproductive biomass and/or seed number rather than the size of individual seed (Venable 1992; Fernández-Pascual *et al.* 2019; Kang *et al.* 2021). However, due to the lack of data on plant growth and reproduction characteristics, we could not determine which factor contributed more to our findings.

We are the first to analyse the relationship between disturbance niche breadth and ITV. Plant species often vary significantly in reproductive strategies to respond to different disturbance levels. For example, many species tend to reproduce early, shorten fruit development time or increase seed number as adaptation to high disturbance frequency and severity (Kühner and Kleyer 2008; Mabry and Fraterrigo 2009; Johnson and Miyanishi 2010; Veloso *et al.* 2017). The variation in reproductive strategies is expected to affect seed mass by varying the reproductive output, pattern of reproductive allocation and the seed mass/number trade-off of individual plant (Wu *et al.* 2015; Herben *et al.* 2018). Thus, the positive interspecific relationship between disturbance niche breadth and ITVsm may imply that species with variable reproductive strategies have the capacity to distribute widely along disturbance gradients. The positive relationship, however, was not supported by phylogenetic analysis, suggesting the cross-species correlation between species disturbance niche breadth and their intraspecific variation in reproductive strategies or seed mass and should be driven mainly by one or more large divergences deep in the phylogeny rather than by a consistent trait association throughout the evolutionary history of the clade.

Both interspecific and phylogenetic analyses showed a significantly lower ITVsm for zoochorous species than others, supporting our expectation that the size variation of zoochorous seeds is restricted because they need to match structurally the size of animal-dispersal appendages and/or seed dispersers’ organ. However, the ITVsm of anemochorous species was not different from that of autochorous species, implying no obvious structural limitation on intraspecific anemochorous seed size variation. Moreover, relative to zoochorous seeds, the dispersal distance of anemochorous seeds was generally short, and determined mainly by wind speed, the shape and type of dispersal appendages and seed mass (Greene and Johnson 1993; Savage *et al.* 2014; Zhou *et al.* 2019). Therefore, a strong seed mass variation helps maternal plants of anemochorous species spread seeds to different locations to avoid sibling competition (Savage *et al.* 2014; Traveset *et al.* 2014; Zhang *et al.* 2020). Surprisingly, we did not find significant difference in ITVsm among life forms. Because life forms differ in plant lifespan, the quality of available resources and the way of partitioning and storing resources (Campanella and Bertiller 2008; Qi *et al.* 2014; Zhang *et al.* 2020), this finding suggests a lack of direct effects of plant lifespan, and resource availability and allocation patterns on the development of individual seed.

The non-significant effect of pollination type on ITVsm may imply little, if any, pollen limitation on reproductive success and seed output and development (Walsh *et al.* 2014), which makes among-population seed mass variation less dependent on pollination vector. In addition, some specific pollination strategies, such as facultative or delayed autogamy in alpine zones (Sun *et al.* 2005; Dainese and Bragazza 2012; Xiong *et al.* 2013), can provide plants with substantial reproductive insurance in cases of low pollinator activity, which further weakens the effect of pollination vector on the difference in seed output among populations.

## Conclusion

These results partly support our expectations. First, the ITVsm–species niche breadth is significantly positive in the habitat-scale light, moisture and disturbance dimensions, but not in the local-scale thermal dimension, suggesting a stronger effect of habitat conditions than locally available resources on the among-population difference in seed mass. Then, seed dispersal mode, but not of life form and pollination type, is the only biotic attributes affecting significantly ITVsm, implying that the covariation or co-evolution between seed and disperser, rather than plant vegetative growth characteristics and reproductive strategies, is strongly related to the pattern and magnitude of ITVsm. Moreover, the multivariate models show significant combined effects of the species biotic attributes and the niche breadth on ITVsm, supporting the idea of a multi-factor control on intraspecific seed development (Sides *et al.* 2014; Fricke *et al.* 2019). Notably, because our study was observational, we cannot conclude to what extent the measured ITVsm represents genetic variation or phenotypic plasticity, but given the higher contribution of the species niche breadth traits (vs. biotic attributes) in ITVsm and a non-significant or weak phylogenetic signal in these traits (light, moisture, disturbance and thermal niche breadth, GCsm and CVsm), it is likely that plasticity plays an important role.

## Supporting Information

The following additional information is available in the online version of this article—

Table S1. Phylogenetic signal for ITVsm (intraspecific seed mass variation) and each of its related biotic attributes and niche breadth traits.

Appendix S1. The methods of seed mass-related biotic attributes and plant niche traits measurement.

Appendix S2. The results of interspecific and phylogenetic analysis that use Gini coefficient value of among-population seed mass variation (GCsm) as the index of intraspecific seed mass variation (ITVsm).

Appendix S3. List of intraspecific seed mass variation, three biotic attributes and four niche breadth traits of 434 angiosperm species. CVsm and GCsm are separately the CV (coefficient of variation) and GC (Gini coefficient) value of among-population seed mass variation. LF, life form; SDM, seed dispersal mode; PT, pollination type; An, annual; Hp, herbaceous perennial; Wp, woody perennial.

Supplementary Material. List of seed mass and seven related biotic attributes and niche traits of 434 angiosperm species (4769 populations). SM, seed mass (mg); LF, life form; SDM, seed dispersal mode; PT, pollination type; LN, light niche; MN, moisture niche; TN, thermal niche; DN, disturbance niche; An, annual; Hp, herbaceous perennial; Wp, woody perennial; Aut, autochory; Ane, anemochory; Ect, ectozoochory; End, endozoochory; Anem, anemophily; Entom, entomophily.

plac013_suppl_Supplementary_Appendix_S1

plac013_suppl_Supplementary_Appendix_S2

plac013_suppl_Supplementary_Appendix_S3

plac013_suppl_Supplementary_Table_S1

plac013_suppl_Supplementary_Material

## Data Availability

The data that support the findings of this study are openly available at: https://figshare.com/s/94672a4f1a8c139791e5.

## Notes

**Populations & Communities. Chief Editor**: Jean Burns
