# Supplementary material for: Regional gradients in intraspecific seed mass variation are associated with species biotic attributes and niche breadth
Source: AoB Plants. 2022 Mar 30;14(2):plac013. doi: 10.1093/aobpla/plac013 (PMC9128389; doi:10.1093/aobpla/plac013)
Supplement: plac013_suppl_Supplementary_Appendix_S1 [file plac013_suppl_Supplementary_Appendix_S1.doc]

The methods of seed mass related biotic attributes and plant niche traits measurement

Species biotic attributes

For every species, we considered three seed mass related biotic attributes, including life form, seed dispersal mode and pollination type. They were categorized as follows.

1. **Life form**. Raunkiaer life form distinguished between annual (therophytes), herbaceous perennial (hemicryptophytes and geophytes), and woody perennial (phanerophytes) (Wu and Raven, 1994-2013). Eight subshrub species (chamaephytes) were treated as herbaceous perennial for their relatively weak woody tissues (Wu and Raven, 1994-2013; Butler et al., 2007; Qi et al., 2014).
2. **Seed dispersal mode**. Dispersal mode was classiﬁed into the following four groups according to the morphological features of their seeds (Wu and Raven, 1994-2013; Leishman et al. 1995; Butler et al., 2007; Qi et al., 2014): autochory (seeds without obvious morphological structure for dispersal), anemochory (seeds with wings, hairs or pappus), ectozoochory (seeds with hooks, spines or barbs), and endozoochory (seeds with an aril or ﬂesh). As ectozoochory and endozoochory are evolved with vertebrates, they were also combined into zoochory.
3. **Pollination type**. As there are scarcely any biotic and abiotic pollen vectors but wind and insects, pollination system was coded as anemophily and entomophily (Wu and Raven, 1994-2013; Qi et al., 2014).

Population niche positions

For every population, we recorded its niche positions (requirements) in light, moisture, disturbance and thermal dimensions. They were measured or categorized as follows.

1. **Light niche position.** Light niche represented the light level of habitat where more than 70% individuals of a population (or more than 70% individuals that were collected seeds) lived (Poorter et al., 2006; Huang et al., 2019). Level 1, extreme shade (about < 5% sunlight, under the thick crown canopy, *etc*); level 2, high shade (about 5%-30% sunlight, under the overgrown shrubbery and copse, under the thick crown canopy with periodic light, *etc*); level 3, moderate shade (about 30%-70% sunlight, under the medium-thick shrubbery and copse, *etc*); level 4, high light (about 70%-95% sunlight, at the edge of shrubbery and copse, in the meadow and grassland with sparse shrubs, *etc*); and level 5, extreme light (about > 95% sunlight, in various open habitats).
2. **Moisture niche position**. Moisture niche position represented the moisture level of habitat where more than 70% individuals of a population (or more than 70% individuals that were collected seeds) occupied (Ellers et al., 2018). Level 1, extreme dryness [mean about < 5% soil moisture content (SMC) in growing season; in desert and other extremely arid habitat, *etc*]; level 2, high dryness (about 5%-15% SMC; in arid savanna-woodlands, arid sparse grassland, *etc*); level 3, neutral moisture (about 15%-40% SMC; in any site without obvious wetness/dryness); level 4, high wetness (about > 40% SMC but no significant surface ponding; in seasonal wetland, waterside, *etc*); and level 5, extreme wetness (> 60% SMC and often with significant surface ponding; in water area, all-year wetland, *etc*).
3. **Thermal niche position**. Referring to the method in Gvoždík (2018) and Ellers et al. (2018), we classified population thermal niche position based on the thermal level (i.e., thermal climatic zone) it survives. Our studied region spans a large climate ranges, from warm-temperature (including north edge of subtropical) [about < 2250 m a.s.l., mean annual temperature (MAT) = 9.6-13.2 °C], cold-temperature (about 2250-2700 m a.s.l., MAT = 6.8-9.6 °C), warm-subalpine (2700-3150 m a.s.l., MAT = 4.0-6.8 °C), cold-subalpine (including the transitional area of subalpine and alpine, 3150-3600 m a.s.l., 1.2-4.0 °C), to alpine (including intermittent tundra) zone (3600-4100 m a.s.l., -1.7-1.2 °C) zones. We treated alpine, cold-subalpine, warm-subalpine, cold-temperature and warm-temperature as thermal levels of 1-5, respectively.
4. **Disturbance niche position**. Disturbance niche position represented the disturbance level (frequency and severity) of habitat where more than 70% individuals of a population (or more than 70% individuals that were collected seeds) occupied (Herben et al., 2018). For the difficulty to quantify habitats’ disturbance frequency and severity, we first divided habitats into three types: high-disturbance habitats (roadsides, farmlands, cut-over areas, old-fields, etc), neutral-disturbance habitats (most grasslands, sparse shrubs and forests with low-intensity artificial or natural disturbance), low- or non-disturbance habitats (closed forests, dense shrubs and other trackless areas). Then, population disturbance niche position was defined as: level 1, lowest disturbance (only live in low- or non-disturbance habitats); level 2, lower disturbance (live in both neutral- and low-disturbance habitats); level 3, neutral disturbance (main live in neutral-disturbance habitats) ; level 4, higher disturbance (live in both high- and neutral-disturbance habitats); level 5, highest disturbance (only live in high-disturbance habitats).

References

Butler, D.W., Green, R.J., Lamb, D., McDonald, W.J.F. and Forster, P.I. 2007. Biogeography of seed-dispersal syndromes, life-forms and seed sizes among woody rain-forest plants in Australia's subtropics. Journal of Biogeography, 34: 1736-1750.

Ellers, J., Berg, M.P., Dias, A.T.C., Fontana, S., Ooms, A. & Moretti, M. 2018. Diversity in form and function: Vertical distribution of soil fauna mediates multidimensional trait variation. Journal of Animal Ecology 87: 933-944.

Gvoždík, L. 2018. Just what is the thermal niche? Oikos 127: 1701-1710.

Herben, T., Klimešová, J. & Chytrý, M. 2018. Effects of disturbance frequency and severity on plant traits: An assessment across a temperate flora. Functional Ecology, 32: 799-808.

Huang, L., Xue, W. & Herben, T. 2019. Temporal niche differentiation among species changes with habitat productivity and light conditions. Journal of Vegetation Science, 30: 438-447.

Leishman, M. R., Westoby, M. & Jurado, E. 1995. Correlates of seed size variation: a comparison among five temperate floras. Journal of Ecology, 83: 517-530.

Poorter, L., Bongers, L. & Bongers, F. 2006. Architecture of 54 moist-forest tree species: traits, trade-offs, and functional groups. Ecology, 87: 1289-1301.

Qi, W., Guo, S., Chen, X., Cornelissen, J., Bu, H., Du, G., Cui, X. Li, W. & Liu, K. 2014. Disentangling ecological, allometric and evolutionary determinants of the relationship between seed mass and elevation: insights from multiple analyses of 1355 angiosperm species on the eastern tibetan plateau. Oikos, 123: 23-32.

Wu, C.Y. & P.H. Raven (eds). 1994-2013. Flora of China. Science Press and Missouri Botanical Garden Press, Beijing and St. Louis.
