# Supplementary material for: Regional gradients in intraspecific seed mass variation are associated with species biotic attributes and niche breadth
Source: AoB Plants. 2022 Mar 30;14(2):plac013. doi: 10.1093/aobpla/plac013 (PMC9128389; doi:10.1093/aobpla/plac013)
Supplement: plac013_suppl_Supplementary_Appendix_S2 [file plac013_suppl_Supplementary_Appendix_S2.docx]

The results of interspecific and phylogenetic analysis that use Gini coefﬁcient valueof among-population seed mass variation (GCsm) as the index of intraspecific seed mass variation (ITVsm)

**Table A1.** Results from GLM (generalized linear models) of various predictors on the Gini coefﬁcient value of intraspecific seed mass variation (GCsm). For binary (Xylophyta, Lifespan, Anemochory, Zoochory and Pollination type) and continuous ((light niche breadth, moisture niche breadth, thermal niche breadth and disturbance niche breadth) predictors, column “B” represented the mean GCsm difference between groups (group “1” minus group “0”) and regression slope, respectively. %SS, percentage of total sum of squares explained. * *P*< 0.05, ** *P*< 0.01, and *** *P*< 0.001, respectively.

| Predictor | B | F | %SS |
| --- | --- | --- | --- |
| ***Models*** | Adjusted R^2^ = 0.180 | | |
| Xylophyta | 0.006 | 0.91 | 0.2 |
| Lifespan | 0.000 | 0.01 | 0.0 |
| Anemochory | 0.014 | 12.42*** | 2.8 |
| Zoochory | -0.019 | 14.60*** | 3.3 |
| Pollination type | -0.001 | 0.04 | 0.0 |
| Light niche breadth | 0.012 | 12.89*** | 3.0 |
| Moisture niche breadth | 0.017 | 22.41*** | 5.0 |
| Thermal niche breadth | 0.001 | 0.25 | 0.1 |
| Disturbance niche breadth | 0.015 | 15.78*** | 3.6 |

**Fig. A1** The linear relationship between the Gini coefﬁcient value of intraspecific seed mass variation (GCsm) and species niche breadth in light (A), moisture (B), thermal (C) and disturbance (D) dimensions. Regression lines are shown for significant (*P*< 0.05) linear relationships.


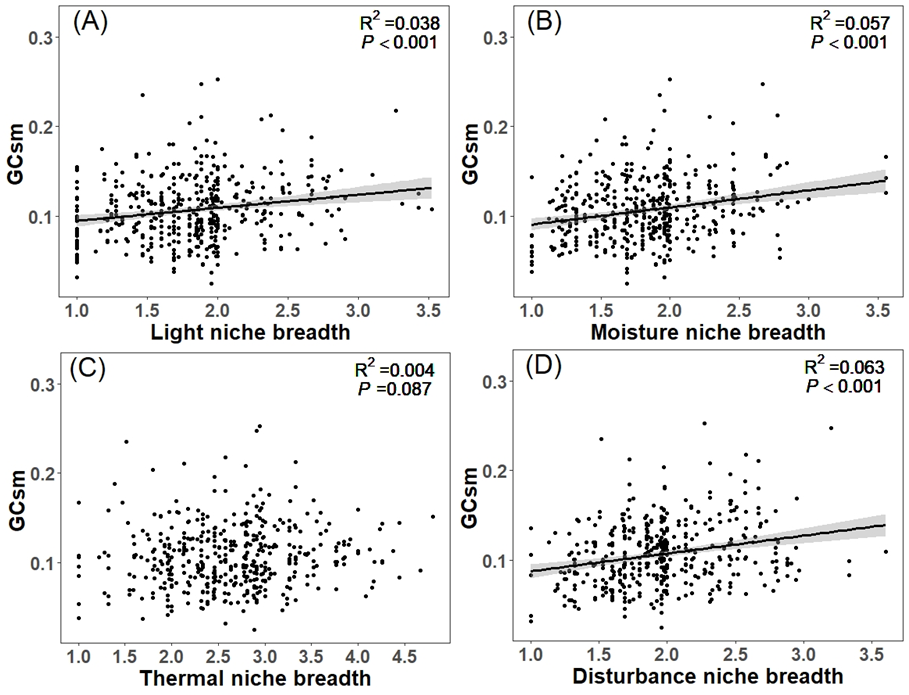


**Fig. A2** Box plot of the variation in GCsm (the Gini coefﬁcient value of intraspecific seed mass variation) with life forms (A), seed dispersal modes (B) or pollination types (C). The ends of the box represent the ﬁrst and third quartiles and the middle line represents the median. The error bars indicate 1.5-fold the inter quartile range. The different lowercase letters indicate signiﬁcant differences of GCsm among life form, seed dispersal mode, or pollination type.


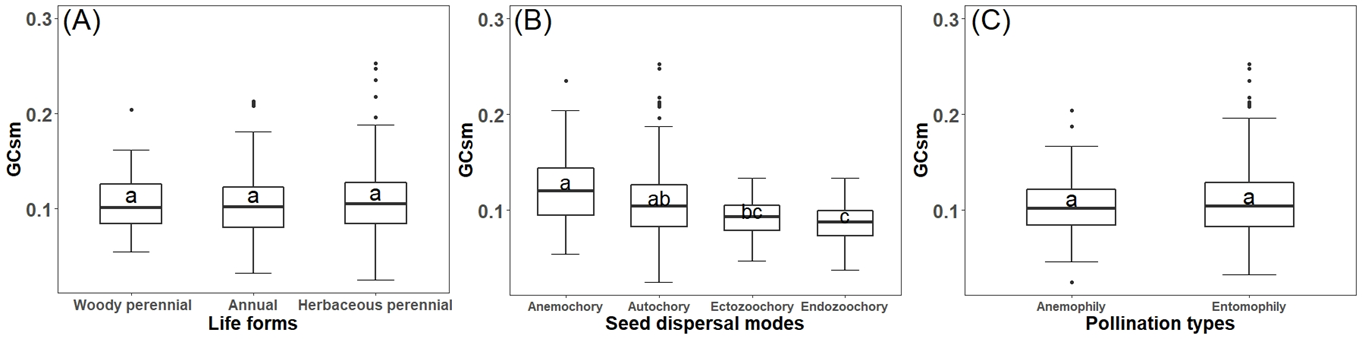


**Fig. A3** Relationship between divergence in GCsm and divergence in light niche breadth (A), moisture niche breadth (B), thermal niche breadth (C) and disturbance niche breadth (D). Abbreviations of GCsm, light niche breadth, moisture niche breadth, thermal niche breadth or disturbance niche breadth are speciﬁed in Figure A1. Regression lines are shown for significant (*P*< 0.05) relationships.


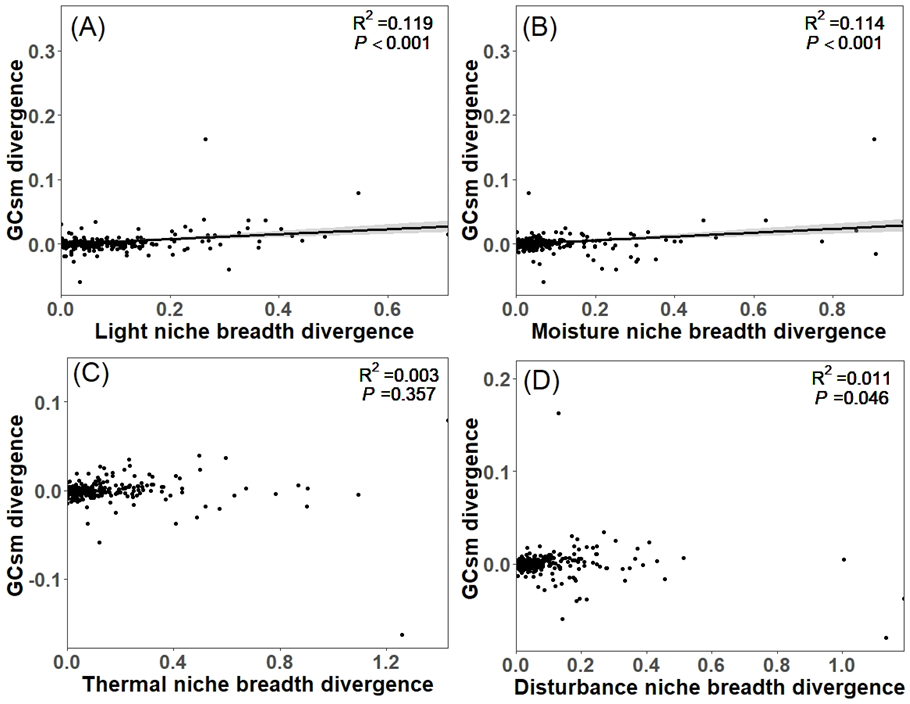


**Fig. A4** Results of the regression tree analysis of the relationship between GCsm (the Gini coefﬁcient value of intraspecific seed mass variation) and seven biotic or niche breadth predictors. In the decision of tree size, 10-fold cross-validations and a minimum child node of ten sampling size are applied, and the Gini index is used as impurity.

Mean = 0.107

S.D. = 0.035

N = 434

>= 1.702

**Disturbance niche breadth**

Mean = 0.112

S.D. = 0.035

N = 304

Mean = 0.094

S.D. = 0.031

N = 130

< 1.702

Mean = 0.116

S.D. = 0.036

N = 259

Mean = 0.092

S.D. = 0.022

N = 45

Mean = 0.110

S.D. = 0.034

N = 147

Mean = 0.124

S.D. = 0.037

N = 112

Anemochory,

Autochory

Zoochory

**Seed dispersal mode**

**Moisture** **niche breadth**

< 1.953

>= 1.953
