# Supplementary material for: Regional gradients in intraspecific seed mass variation are associated with species biotic attributes and niche breadth
Source: AoB Plants. 2022 Mar 30;14(2):plac013. doi: 10.1093/aobpla/plac013 (PMC9128389; doi:10.1093/aobpla/plac013)
Supplement: plac013_suppl_Supplementary_Table_S1 [file plac013_suppl_Supplementary_Table_S1.docx]

**Table S1.** Results of phylogenetic signal, including Pagel's λ and their associated LR (likelihood ratio test) of λ = 0 and *P*-values, for CVsm, GCsm and each of their related biotic and niche breadth traits.

| Traits | Pagel's λ | LR of λ = 0 | *P*-values |
| --- | --- | --- | --- |
| CVsm | 0.1404 | 3.461 | 0.063 |
| GCsm | 0.1203 | 3.191 | 0.074 |
| Xylophyta | 0.9999 | 458.45 | 1.044 * e^-101^ |
| Lifespan | 0.4936 | 110.051 | 9.549 * e^-26^ |
| Anomochory | 0.8805 | 431.606 | 7.268 * e^-96^ |
| Zoochory | 0.8166 | 142.466 | 7.691 * e^-33^ |
| Pollination type | 0.9999 | 1288.070 | 4.430 * e^-282^ |
| Light niche breadth | 0.4435 | 25.818 | 3.753* e^-7^ |
| Moisture niche breadth | 0.2786 | 26.177 | 3.115 * e^-7^ |
| Thermal niche breadth | < 0.0001 | < 0.001 | 1 |
| Disturbance niche breadth | < 0.0001 | < 0.001 | 1 |
